# Effect of Quercetin 3-*O*-β-D-Galactopyranoside on the Adipogenic and Osteoblastogenic Differentiation of Human Bone Marrow-Derived Mesenchymal Stromal Cells

**DOI:** 10.3390/ijms21218044

**Published:** 2020-10-28

**Authors:** Jung Hwan Oh, Fatih Karadeniz, Youngwan Seo, Chang-Suk Kong

**Affiliations:** 1 Marine Biotechnology Center for Pharmaceuticals and Foods, College of Medical and Life Sciences, Silla University, Busan 46958, Korea; wjdghks0171@naver.com (J.H.O.); karadenizf@outlook.com (F.K.); 2 Division of Marine Bioscience, Korea Maritime and Ocean University, Busan 49112, Korea; ywseo@kmou.ac.kr; 3 Department of Food and Nutrition, College of Medical and Life Sciences, Silla University, Busan 46958, Korea

**Keywords:** adipogenesis, mesenchymal stromal cell, osteoblastogenesis, quercetin, Wnt/β-catenin

## Abstract

Natural products, especially phenols, are promising therapeutic agents with beneficial effects against aging-related complications such as osteoporosis. This study aimed to investigate the effect of quercetin 3-*O*-β-D-galactopyranoside (Q3G), a glycoside of a common bioactive phytochemical quercetin, on osteogenic and adipogenic differentiation of human bone marrow-derived mesenchymal stromal cells (hBM-MSCs). hBM-MSCs were induced to differentiate into osteoblasts and adipocytes in the presence or absence of Q3G and the differentiation markers were analyzed to observe the effect. Q3G treatment stimulated the osteoblastogenesis markers: cell proliferation, alkaline phosphatase (ALP) activity and extracellular mineralization. In addition, it upregulated the expression of RUNX2 and osteocalcin protein as osteoblastogenesis regulating transcription factors. Moreover, Q3G treatment increased the activation of osteoblastogenesis-related Wnt and bone morphogenetic protein (BMP) signaling displayed as elevated levels of phosphorylated β-catenin and Smad1/5 in nuclear fractions of osteo-induced hBM-MSCs. The presence of quercetin in adipo-induced hBM-MSC culture inhibited the adipogenic differentiation depicted as suppressed lipid accumulation and expression of adipogenesis markers such as PPARγ, SREBP1c and C/EBPα. In conclusion, Q3G supplementation stimulated osteoblast differentiation and inhibited adipocyte differentiation in hBM-MSCs via Wnt/BMP and PPARγ pathways, respectively. This study provided useful information of the therapeutic potential of Q3G against osteoporosis mediated via regulation of MSC differentiation.

## 1. Introduction

Osteoporosis is a common medical condition in which bone formation becomes imbalanced, and the bone is filled with adipocytes rather than osteoblasts [1]. This imbalance in the bone mass manifests as increased fragility and prone to fractures. To date, osteoporosis has been treated with a wide range of treatments targeting different mechanisms of bone buildup and employing different molecules such as bisphosphonates, parathyroid hormone, and selective estrogen receptor modulators. Over the years, studies have shown that the differentiation tendencies of the bone marrow mesenchymal stem cells (MSCs) affect the composition of bone directly [2]. The reciprocal relationship between the adipogenesis and osteoporosis of MSCs makes inducing MSCs in favor of osteoblastogenesis to increase bone mass a promising therapeutic target for osteoporosis treatment. Regulation of MSC differentiation occurs through different pathways critically intertwined between adipogenesis and osteoblastogenesis [3]. When the cells are induced to differentiate into one lineage, inducers often suppress the differentiation into others. Studies have identified two pathways that regulate MSC differentiation where one is activated, the other is suppressed: peroxisome proliferator-activated receptor γ (PPARγ) and Wnt/β-catenin signaling [4]. Inducing MSCs to differentiate into adipocytes through PPARγ activation inhibits the bone morphogenetic protein (BMP)-induced osteoblastogenesis and vice versa.

Most of natural products with pharmaceutical potential against metabolic syndrome-linked complications are of plant origin. These substances—including, but not limited to, flavanoids, coumarins, and terpenes—have been shown to possess therapeutic properties for the symptoms of obesity, diabetes, cardiovascular diseases, and osteoporosis [5,6]. Different types of flavonoids, a very common polyphenol secondary metabolite found in plants and fungus, are included in several commercial preparations with medical, nutritional, and cosmeceutical uses [7]. Studies have reported that some of these flavonoids exert anti-adipogenic properties via different mechanisms of action depending on the chemical structure of the compound [8,9]. Quercetin is a such flavonoid from flavonol sub-class and one of the most common flavonoids in human daily diet. Quercetin is primarily found in its glycoside form distributed among various plants as well as plant products, such as wine, tea and medicinal preparations [10]. Quercetin and its derivatives are known for their high bioavailability and good free radical scavenging properties [11]. Nutritional benefits of quercetin derivatives vary from improved cardiovascular health to reduced risk of tumor progression and skin aging [12,13,14].

**Figure 1:**
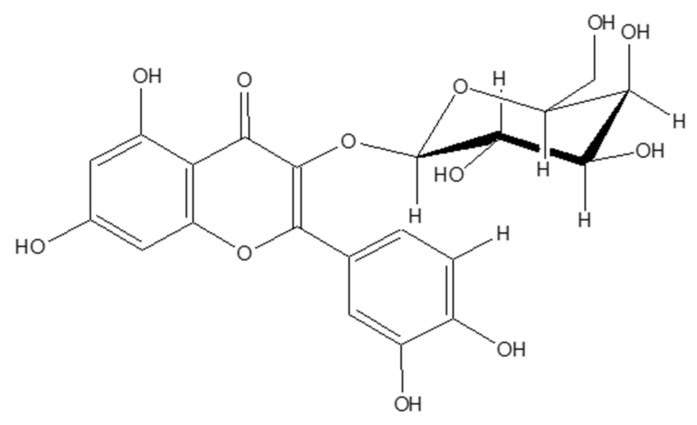
Chemical structure of quercetin-3-*O*-β-D-galactopyranoside (Q3G).

Studies have showed that not only quercetin, but also its derivatives, showed promising health beneficial effects [11]. In this context, several derivatives of the quercetin screened for their potential anti-adipogenic and pro-osteogenic activities. Therefore, the current study aimed to evaluate the anti-osteoporotic potential of quercetin-3-*O*-β-D-galactopyranoside (Q3G) (Figure 1), a quercetin glycoside, via adipogenic and osteogenic differentiation of human bone marrow-derived mesenchymal stromal cells (hBM-MSCs). To the best of our knowledge, Q3G has not been yet studied for its anti-adipogenic and pro-osteogenic activities in hBM-MSCs. The results of current study were suggested to provide insights towards potential use of Q3G as a preventive natural product against osteoporotic complications.

## 2. Results

### 2.1. Proliferation, ALP Activity and Extracellular Mineralization of Osteo-Induced hBM-MSCs

**Figure 2:**
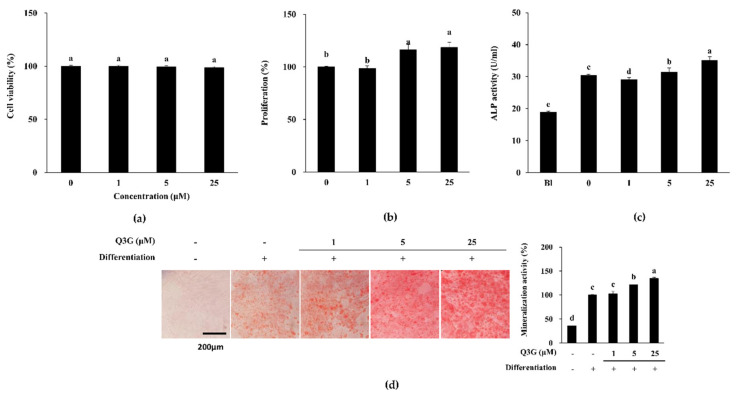
Effect of Q3G on the osteoblastogenic differentiation of hBM-MSCs. (**a**) Effect of Q3G on on the viability of non-induced human bone marrow-derived mesenchymal stromal cells (hBM-MSCs). Viability of the cells was measured by quantification of MTT dye removed from cells after 48 h of treatment. Cell viability is given as relative percentage of untreated control. (**b**) Effect of Q3G on the proliferation of osteo-induced hBM-MSCs. Viable cell amount was measured by quantification of MTT dye removed from cells at day 3 of differentiation. Proliferation was given as relative viable cell amount (%) of untreated osteo-induced control. (**c**) Effect of Q3G on the activity of cellular alkaline phosphatase (ALP). Cellular ALP activity of osteo-induced hBM-MSCs was measured with a spectrophotometric enzymatic activity assay at day 7 of differentiation. Q3G was present in the first 3 days of differentiation only. (**d**) Effect of Q3G on the extracellular mineralization of osteo-induced hBM-MSCs. Extracellular mineralization was measured by Alizarin Red staining and quantified by the absorbance values of the retained dye at day 10 of differentiation. Mineralization was given as a relative percentage of untreated osteo-induced hBM-MSCs. Q3G was treated in the first 3 days of differentiation only. Different letters (a–e) indicate statistically significant difference (*p* < 0.05) among groups.

Q3G did not exert any cytotoxicity in non-induced hBM-MSCs following 72 h of treatment up to concentration of 25 μM (Figure 2a). After 72 h, viable cell amount was at the same level with untreated group for all Q3G-treated groups. However, the presence of Q3G in the initial differentiation medium introduction increased the viable cell amount starting from 5 μM treatment (Figure 2b). At 25 μM concentration, the Q3G-treated group contained an 18.81% higher cell count compared to non-treated osteo-induced hBM-MSCs.

At day 10 of differentiation, samples from osteo-induced hBM-MSCs without Q3G treatment expressed 30.51 U/mL alkaline phosphatase (ALP) activity whereas non-induced untreated hBM-MSC samples expressed 18.91 U/mL (Figure 2c). When hBM-MSCs were exposed to Q3G for 3 days at the beginning of the differentiation, ALP activity was increased to 31.64 U/mL for 5 μM and 35.17 U/mL for 25 μM. On the contrary, 1 μM Q3G treatment caused a slight decrease in ALP activity (29.13 U/mL). On the other hand, Q3G treatment stimulated extracellular mineralization compared to untreated osteo-induced hBM-MSCs in a dose-dependent manner revealed by Alizarin Red staining (Figure 2d). At 25 μM, Q3G-treated osteo-induced hBM-MSCs displayed 34.43% more calcification than that of untreated hBM-MSCs.

### 2.2. Expression of Osteoblastogenesis Marker Genes and Proteins

**Figure 3:**
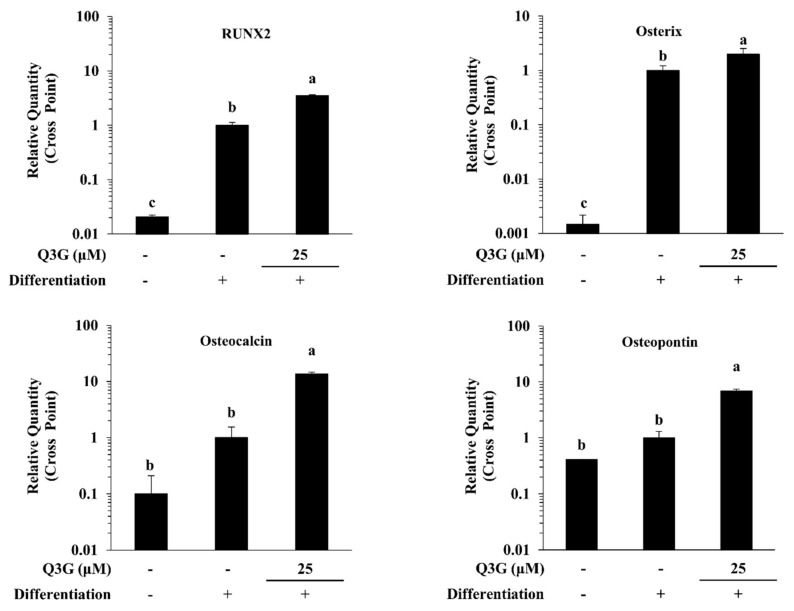
Effect of Q3G on osteoblastogenesis marker gene expression. Analysis of gene expression was carried by measuring mRNA levels of osteo-induced hBM-MSCs via RT-qPCR at day 10 differentiation. Values are normalized against β-actin as internal loading control. Osteo-induced hBM-MSCs were treated with Q3G until day 3 of differentiation. Different letters (a–c) indicate statistically significant difference (*p* < 0.05).

**Figure 4:**
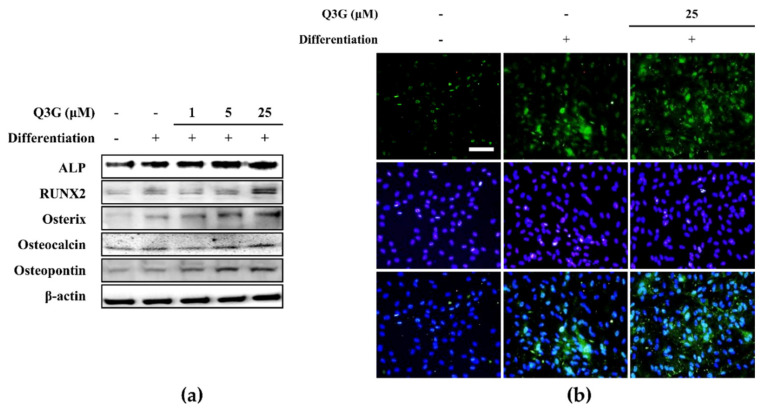
Effect of Q3G on osteoblastogenesis marker protein expression. Analysis of protein expression was carried out by Western blotting (**a**) and immunofluorescence staining (**b**) of osteo-induced hBM-MSCs at day 10 differentiation. Q3G was treated with initial differentiation induction (3 days) and Q3G was not present in subsequent media changes. β-actin was used as internal loading control. Scale bar: 100 μm.

The effect of Q3G on osteoblastogenic differentiation of hBM-MSCs was further observed in gene and protein expression levels. At day 10 of differentiation, the osteo-induced hBM-MSCs expressed significantly high levels of RUNX2, an osteoblastogenesis marker gene, compared to non-induced cells. As its downstream effectors for osteoblast maturation, the mRNA levels of osterix, osteocalcin and osteopontin were also increased with inducing osteoblastogenesis (Figure 3). The presence of 25 μM Q3G in the initial differentiation medium further increased the mRNA levels of RUNX2, osterix (pre-osteoblast marker), osteocalcin (differentiated osteoblast marker) and osteopontin (differentiated osteoblast marker). Similar outcomes were observed for the protein levels of osteoblastogenesis markers following Q3G treatment. In a dose-dependent manner, protein levels of RUNX2, osteorix, osteocalcin and osteopontin as well as ALP were increased by Q3G treatment compared to untreated osteo-induced hBM-MSCs (Figure 4a). The effect of Q3G on the expression of early osteoblastogenesis markers was also investigated by fluorescence staining of the RUNX2 protein in differentiated hBM-MSCs. Expectedly, cells treated with 25 μM Q3G displayed increased amounts of RUNX2 protein (Figure 4b).

### 2.3. Effect of Q3G on Wnt/BMP Signaling

**Figure 5:**
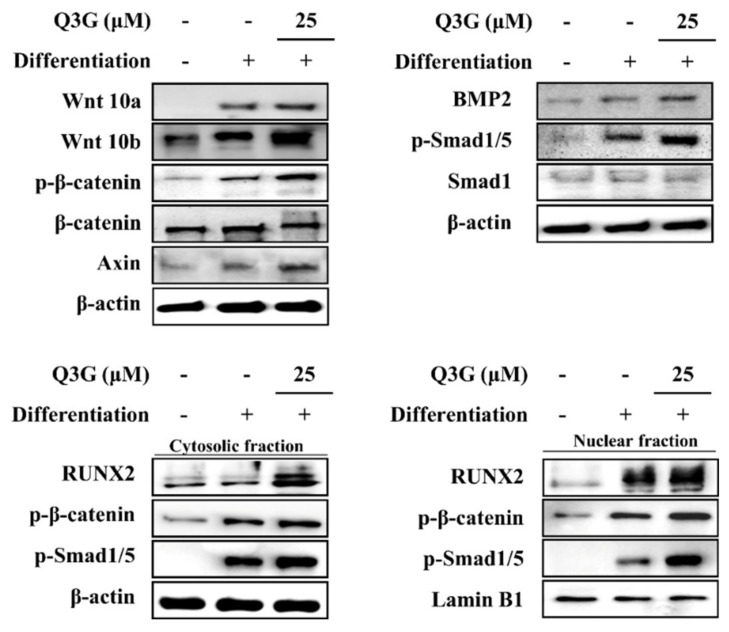
Effect of Q3G on Wnt/β-catenin and BMP signaling pathways. Analysis of protein expression was carried out by Western blotting of osteo-induced hBM-MSCs at day 10 differentiation. Q3G was treated with initial differentiation induction (3 days) and it was not present in subsequent media changes. β-actin (for whole cell and cytosolic fraction) and lamin B1 (for nuclear fraction) were used as internal loading control.

Activation of RUNX2 cascade to initiate osteoblastogenesis in osteoprogenitor cells was examined through the expression and phosphorylation of canonical Wnt/BMP signaling. Osteo-induced hBM-MSCs expressed elevated Wnt10a and Wnt10b levels (Figure 5). This elevation was accompanied by elevated phosphorylation of β-catenin. In addition, free axin levels were also elevated as a result of increased β-catenin levels due to axin being a part of the β-catenin destructive complex. Similar results were obtained from BMP2 signaling. Osteo-induction increased the expression of BMP2 levels as well as phosphorylation of its downstream effector Smad1/5 complex in osteo-induced hBM-MSCs (Figure 5). The levels of Wnt10a, Wnt10b and BMP2 were enhanced by the treatment with Q3G (25 μM) compared to those of the untreated osteo-induced hBM-MSCs. As expected, Q3G was also able to induce further activation of β-catenin and Smad1/5 complex.

The cytosolic and nuclear levels of RUNX2 protein, expressed as a result of the β-catenin and Smad1/5 complex nuclear translocation, were increased with osteoblastogenic differentiation which was further enhanced by Q3G treatment (25 μM) (Figure 5). Both nuclear and cytosolic fractions of Q3G-treated hBM-MSCs expressed significantly higher levels of RUNX2, as well as β-catenin and Smad1/5 complex.

### 2.4. Lipid Accumulation of Adipo-Induced hBM-MSCs

**Figure 6:**
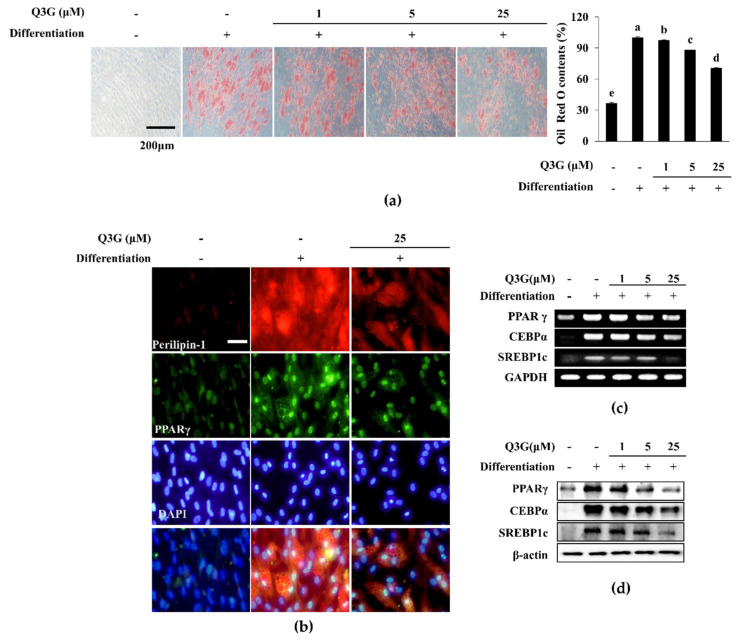
Effect of Q3G on adipogenic differentiation of hBM-MSCs. (**a**) Effect of Q3G on the intracellular lipid accumulation in adipo-induced hBM-MSCs at day 10 of differentiation. Lipid droplets were stained with Oil Red O and the quantification was carried out by measuring absorbance values of retained dye. Lipid accumulation level was given as relative percentage of Oil Red O dye compared to adipo-induced untreated group. Different letters (a–e) indicate statistically significant difference (*p* < 0.05) among groups. (**b**) Effect of Q3G on the expression of perilipin-1 and PPARγ in adipo-induced hBM-MSCs at day 10 differentiation analyzed by immunofluorescence staining. DAPI staining was used to highlight the nucleus of viable cells. Scale bar: 50 μm. Effect of Q3G on the expression of adipogenic marker genes (**c**) and proteins (**d**). Analysis of gene and protein expression of adipo-induced hBM-MSCs was carried out by RT-PCR and Western blot, respectively, at day 10 of differentiation. GAPDH and β-actin were used as internal loading controls. Q3G was treated with initial differentiation for 3 days.

Adipo-induced hBM-MSCs displayed adipocyte characteristics after 10 days of differentiation shown by Oil Red O staining of the intracellular lipid droplets (Figure 6a). The addition of Q3G to that initial differentiation medium resulted in a dose-dependent decrease in lipid accumulation in adipo-induced hBM-MSCs, suggesting that it hindered adipogenic differentiation. At 25 μM, Q3G-treated hBM-MSCs accumulated 29.69% less lipid compared to untreated adipo-induced hBM-MSCs. Adipogenic differentiation-induced lipid accumulation was further investigated through fluorescence staining of the perilipin-1 protein, a protein that outlines the intracellular lipid droplets. Similar to Oil Red O staining, Q3G treatment significantly decreased the perilipin-1 levels in adipo-induced hBM-MSCs, which was apparent after inducement compared to non-induced cells (Figure 6b).

### 2.5. Expression of Adipogenesis Marker Genes and Proteins

Adipogenic differentiation of induced hBM-MSCs was also assessed by measuring the mRNA and protein expression of adipogenic markers: PPARγ, CEBPα and SREBP1c. Prior to evaluating mRNA and protein levels, PPARγ levels were measured with fluorescence staining. Adipo-induced hBM-MSCs expressed significantly less nuclear PPARγ levels which was revealed as decreased fluorescence intensity following Q3G treatment (Figure 6b). On the other hand, the inhibitory effect of Q3G on the adipogenic differentiation of hBM-MSCs was also observed in mRNA and protein expression of adipogenic markers. Treating adipo-induced hBM-MSCs with 25 μM Q3G decreased the expression of mRNA and protein levels of PPARγ, CEBPα and SREBP1c, which were significantly stimulated with adipogenic induction (Figure 6c,d).

### 2.6. Effect of Q3G on MAPK/AP-1 Signaling

**Figure 7:**
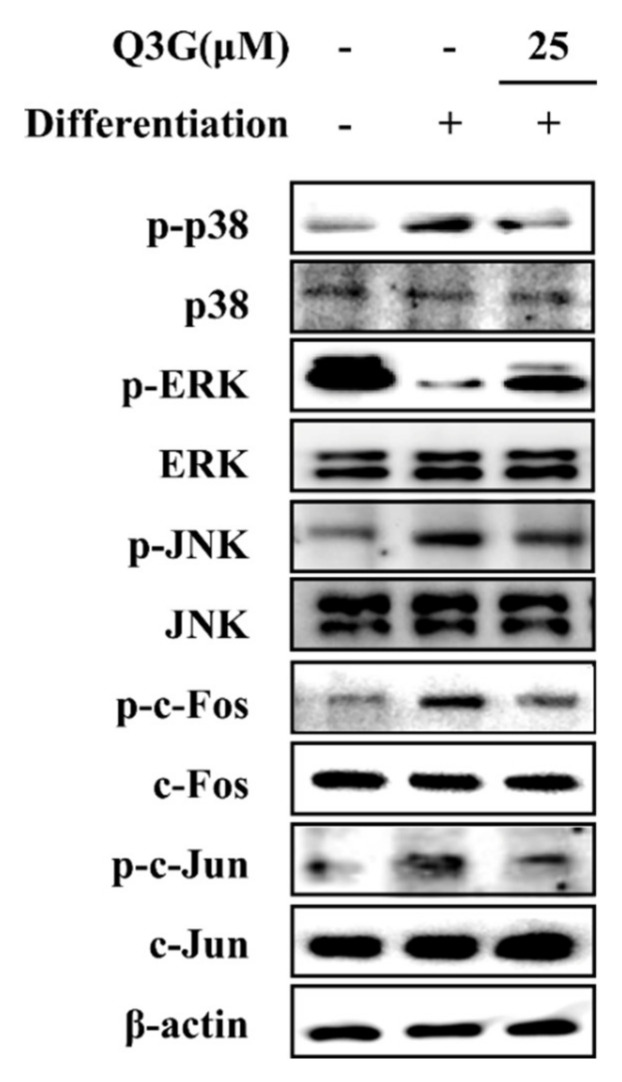
Effect of Q3G on the MAPK/AP-1 signaling. Analysis of MAPK and AP-1 activation and was carried out with Western blotting of whole cell lysates of adipo-induced hBM-MSCs at day 10 of differentiation. Q3G was treated with initial differentiation for 3 days. β-actin was used as internal loading control.

In order to evaluate the mechanism behind the effect of Q3G on adipogenic differentiation of hBM-MSCs, MAPK/AP-1 signaling was examined. Inducing hBM-MSCs to adipogenesis resulted in elevated phosphorylation of p38 and JNK MAPKs and reduced phosphorylation of ERK at day 10 of differentiation. The presence of 25 μM Q3G reverted the adipogenic differentiation-induced changes in p38, ERK and JNK phosphorylation (Figure 7). Nuclear fractions of adipo-induced hBM-MSCs showed decreased phosphorylation levels for c-Fos and c-Jun (sub-components of AP-1) after Q3G treatment, suggesting that it decreased the adipogenic differentiation via suppression of MAPK-regulated AP-1 activity.

## 3. Discussion

Nutraceuticals derived from plant phenolic substances are credited for various beneficial effects towards the preventive approach against common diseases and disorders such as diabetes, cancer, osteoporosis, obesity, and cardiovascular complications [5,6,15,16]. Quercetin is a popular and widely researched phytochemical in this sense, with reported bioactivities [17,18]. It holds the potential to yield lead compounds to be utilized as nutraceuticals against different syndromes. However, to promote any quercetin derivative as a potential natural product, its biological activity, action mechanism and drawbacks need to be analyzed in detail. In this context, the current study evaluated the effect of a quercetin glycoside, Q3G, on the adipogenic and osteogenic differentiation of hBM-MSCs in order to provide insights for its anti-osteoporotic potential.

It has been shown that quercetin has a beneficial effect on bone resorption, osteogenic differentiation and overall bone repair mechanisms [19,20]. Some reports displayed that quercetin treatment enhanced the osteoblastogenic differentiation of pre-osteoblasts through increased ALP activity and mineralization [21]. On the contrary, it has also been shown that quercetin presence might inhibit the pre-osteoblast proliferation and osteoblast maturation [22]. The differences between the organism of the cells, quercetin concentration and treatment period were found to be strongly regulative on the outcome of treatment. While treating osteo-induced cells with quercetin during the early stages of osteogenesis enhanced the osteoblast maturation, the presence of quercetin in the whole differentiation process resulted in inhibited osteoblast differentiation [23]. Current results reported that treating with Q3G (up to 25 μM) only during the first 3 days of differentiation increased the mineralization and proliferation of osteo-induced hBM-MSCs. This agreed with other studies where quercetin and its derivatives enhanced osteoblast differentiation when added to the initial differentiation medium and not present in further differentiation processes [21,24]. This suggested that the beneficial effect of Q3G was due to its effects on the early osteoblastogenesis mechanism: i.e., the transcriptional activities of RUNX2 and other osteoblast marker transcription factors. The elevated levels of both mRNA and protein expression of RUNX2, osterix, and osteoblast-specific proteins (expressed as a result of RUNX2-mediated transcriptional activity) osteopontin and osteocalcin were all significantly elevated in the Q3G-treated group compared to untreated osteoblasts. 

Potentially, Q3G-mediated osteoblastogenesis enhancement occurred through canonical Wnt pathway via β-catenin and BMP signaling. These signaling cascades regulate the osteoblast differentiation via RUNX2 which was enhanced by Q3G treatment. The present study showed that presence of Q3G in the differentiation medium increased the expression of Wnt10a and Wnt10b of the Wnt pathway, and BMP2 of the BMP pathway. The Wnt10a and Wnt10b were reported to play roles in selective differentiation of MSCs by inhibiting adipogenesis and tilting the differential tendencies of MSCs towards osteogenesis [25,26]. Expectedly, Q3G-induced enhancement of Wnt10a and Wnt10b expression resulted in elevated phosphorylation of β-catenin, downstream effectors of Wnt signaling. Similar results were obtained for the phosphorylation of BMP2 downstream effector Smad1/5/8 complex. The nuclear translocation of phosphorylated Smad1/5 positively regulated the transcriptional activities of RUNX2 [27]. Nuclear fractions of Q3G-treated osteo-induced hBM-MSCs exerted increased levels of β-catenin and Smad1/5 compared to untreated osteoblast. This indicated that Q3G stimulated osteoblast differentiation via canonical Wnt and BMP pathways which resulted in increased RUNX2 activity. Interestingly, quercetin was reported to inhibit Wnt/β-catenin signaling in pluripotent carcinoma cells [28]. Moreover, high concentrations of quercetin reduced the nuclear β-catenin levels in osteo-induced MSCs [23]. However, the current results indicated that Q3G acted opposite in osteo-induced hBM-MSCs. Glycosidic derivatization of quercetin might be responsible for this different effect as well as the difference between treatment periods and in vitro models. Nevertheless, the current study showed that Q3G showed positive effects on osteoblast differentiation of hBM-MSCs in relatively low doses (up to 25 μM).

Increased adipogenic differentiation of bone marrow MSCs opposed to diminished osteogenic differentiation is observed in osteoporosis [2]. Hence, Q3G was also analyzed for its effect on adipogenic differentiation. The primary characteristic of white adipocytes is the intracellular accumulation of lipids. Treating adipo-induced hBM-MSCs with Q3G inhibited the lipid accumulation in adipo-induced hBM-MSCs. This suggested that Q3G might suppress adipogenesis. To confirm its effect on adipogenesis, adipogenic marker genes were checked under Q3G treatment. The main adipogenic transcription factor, PPARγ [29], was inhibited with Q3G treatment, confirming the suppressing effect on adipogenic differentiation. Along with PPARγ, early adipogenesis transcription factors SREBP1c and C/EBPα [30] were also inhibited by Q3G, further suggesting its inhibitory effect on adipogenesis. The PPARγ gene expression is partly regulated by activities of AP-1 which is activated as a downstream protein of MAPK signaling [31]. In the current study, adipo-inducement of hBM-MSCs resulted in increased phosphorylation of p38 and JNK MAPKs and reduced ERK phosphorylation. This was expected, because ERK1/2 negatively regulates the adipogenic maturation during later stages of adipogenesis [32]. Q3G treatment reverted the adipogenesis-induced changes in these MAPKs. This indicated that Q3G might suppress the adipogenic differentiation via inhibiting the transcriptional activities of PPARγ. On the contrary, in another report, a high concentration of quercetin was reported to induce adipogenesis in pre-adipocytes via increasing the activation of PPARγ signaling [23]. However, current results showed that, as opposed to activities of quercetin, Q3G inhibited adipogenic differentiation. As stated earlier, this might be due to its structural differences with quercetin or in this case due to the difference between in vitro models, as the adipogenic stimulatory effects of quercetin were only reported in pre-adipocytes and cells of other organisms other than those of humans.

In addition, Wnt/β-catenin and PPARγ are antagonistic pathways in MSC differentiation, where activation of osteogenic differentiation via Wnt signaling subsequently suppresses PPARγ [33]. It has been reported that the Wnt/β-catenin pathway is an important signaling cascade in the treatment of osteoporotic complications [34]. Moreover, Jing et al. [35] suggested that shifting the MSC differentiation from adipogenesis to osteoblastogenesis via Wnt and PPARγ pathways effectively attenuated the osteoporosis-induced deterioration in bone formation. Positive regulation of Wnt signaling by Q3G is in accordance with its effect on PPARγ pathway. This suggested that Q3G enhanced osteogenic differentiation in osteo-induced hBM-MSCs while inhibiting adipogenesis following adipo-inducement via linked pathways of Wnt and PPARγ, respectively. Quercetin was reported to show varying results on adipogenic and differentiation of MSCs, mainly depending on the dose and treatment stage [23]. In this context, the effect of Q3G would provide insights for future studies to develop novel anti-osteoporotic agents or to overcome the shortcomings of quercetin via diversification.

In conclusion, treating induced hBM-MSCs with Q3G enhanced their osteoblast differentiation and inhibited adipocyte differentiation. Q3G stimulated Wnt/BMP signaling to increase osteoblast formation in osteo-induced hBM-MSCs, whereas it suppressed MAPK-regulated PPARγ activities during adipogenesis. The results suggest that, although further in vivo and detailed mechanistic studies are needed, Q3G is a potential natural substance with beneficial effects on bone formation which can help to develop natural products and therapeutic strategies against osteoporosis.

## 4. Materials and Methods

### 4.1. Q3G Isolation and Characterization

Q3G has been isolated from *Limonium tetragonum* and characterized by ^1^H and ^13^C NMR and comparison with published literature as detailed in an earlier report [36].

### 4.2. Cell Culture and Differentiation

Bone marrow-derived human mesenchymal stem cells (hBM-MSC) were procured from PromoCell (cat. no. C-12974; PromoCell, Heidelberg, Germany). Cells were seeded in 6-well plates (1 × 10^6^ cells/well) and cultured using Mesenchymal Stem Cell Growth Medium (cat. no. C-28009; PromoCell). Incubation of the plates was carried out in an environment with 37 °C temperature and 5% CO_2_ atmosphere. For adipogenic differentiation of hBM-MSC, cells were grown to confluence prior to swapping cell culture medium with Mesenchymal Stem Cell Adipogenic Differentiation Medium 2 (cat. no. C-28016; PromoCell). Following the introduction of differentiation medium, cells were incubated for 10 days (unless otherwise noted). The cell culture medium was changed every third day without disturbing cell monolayer. Q3G was supplied along initial differentiation induction and was not present in consequent media changes. In the case of osteogenic differentiation of hBM-MSCs, same process was carried out replacing the differentiation-inducing medium with Mesenchymal Stem Cell Osteogenic Differentiation Medium (cat. no. C-28013; PromoCell).

### 4.3. Cell Viability Assay

The effect of Q3G on the viability of hBM-MSCs was investigated using common MTT assay procedures. Cells were seeded in 96-well plates (1 × 10^3^ cell/well) and incubated for 24 h. The incubation was followed by the sample treatment. Viability of the treated and untreated cells were quantified after 24 h incubation. Briefly, wells were aspirated after 24 h treatment and were supplied with 100 μL of MTT reagent (1 mg/mL). Plates were then kept under dark for 4 h at room temperature. Viable cell-dependent formation of formazan salts was quantified by addition of 100 μL DMSO to each well and measurement of the absorbance value at 540 nm with a microplate reader (Multiskan GO, Tecan Austria GmbH, Grodig, Austria).

### 4.4. Oil Red O Staining

Display of the accumulated intracellular lipid droplets by adipocytes was carried out with common Oil Red O staining protocols. Briefly, cells were cultured in 6-well plates and differentiated into adipocytes as previously described. Following 10 days of differentiation, wells were aspirated and washed with PBS followed by cell fixation via addition of 10% fresh formaldehyde (in PBS, *v*/*v*). Fixation was continued for 1 h at room temperature. Staining of the lipid droplets was performed by the addition of 1 mL Oil Red O solution (prepared in 6 parts of isopropanol and 4 parts of water) into aspired and washed (with PBS) wells. After 1 h, Oil Red O staining solution was removed, and wells were air-dried. Stained lipid droplets were visualized under an optical microscope (Olympus, Tokyo, Japan). Stain from the lipid droplets was eluted by the presence of 100% isopropyl alcohol in wells. Quantification was carried out by colorimetry measuring the absorbance of the wells (containing retained dye and 100% isopropyl alcohol) at 500 nm using a microplate reader (MultiSkan GO).

### 4.5. Reverse Transcription-Semi-Quantitative PCR

The hBM-MSCs were grown to confluence in 6-well plates (1.5 × 10^6^ cells/well), induced to differentiate as stated earlier. Total RNA was obtained from differentiated hBM-MSC adipocytes using Trizol reagent (Invitrogen, CA, USA) at day 10 of differentiation. Total RNA (2 μg) was reverse transcribed into cDNA using oligo(dT) in RNase-free water and a T100thermocycler (Bio-Rad Laboratories, Inc., Hercules, CA, USA) with an initial denaturation at 70 °C for 5 min followed by immediate cooling. Subsequently, a master mix was prepared containing 1X RT buffer, 1 mM dNTPs, 500 ng oligo(dT), 140 U M-MLV reserve transcriptase and 40 U RNase inhibitor. The following temperature protocol was used for reverse transcription: 42 °C for 60 min and 72 °C for 5 min. Subsequently, qPCR was performed using the primers reported earlier [35]. The following thermocycling conditions were used for qPCR: 30 cycles of 95 °C for 45 sec, 60 °C for 1 min and 72 °C for 45 sec. The final PCR products were separated by electrophoresis for 30 min at 100 V on a 1.5% agarose gel. Following staining with 1 mg/mL ethidium bromide, gels were imaged under a UV light using a CAS-400SM Davinch-Chemi Imager™ (Davinch-K).

### 4.6. Immunoblotting

Detection of protein levels was performed using standard Western blot protocols. Briefly, cells in 6-well plates treated with or without samples were lysed by the addition of 1 mL RIPA buffer and vigorous pipetting at day 10 of differentiation. Cell lysates were assessed for their protein content with a BCA protein assay kit (Thermo Fisher Scientific). A part of total protein lysate (20 μg) was separated by SDS-PAGE (4% stacking and 10% separating gels). Proteins on gels were transferred to polyvinylidene fluoride membrane (Amersham Bioscience., Westborough, MA, USA) for immunoblotting. Blocking of membranes was carried out by keeping membranes in 5% skim milk (*v*/*v* in TBS-T buffer) for 4 h on a shaking incubator. Blocked membranes were hybridized at 4 °C overnight with antibodies against PPARγ (#2443; Cell Signaling Technology, Danvers, MA, USA), CCAAT/enhancer-binding protein (C/EBP)α (#2295; Cell Signaling Technology), sterol regulatory element-binding protein-1c (SREBP-1c) (ab3259; Abcam, Cambridge, England, UK), p38 (#8690; Cell Signaling Technology), phospho(p)-p38 (#4511; Cell Signaling Technology), JNK (LF-PA0047; Thermo Fisher Scientific), p-JNK (sc-293136; Santa Cruz Biotechnology, Santa Cruz, CA, USA), ERK (#4695; Cell Signaling Technology), p-ERK (#4370; Cell Signaling Technology), AMPK (#2603; Cell Signaling Technology), p-AMPK (#2531; Cell Signaling Technology)and β-actin (sc-47778; Santa Cruz Biotechnology) diluted as suggested by the manufacturer in 1X TBST buffer containing 5% bovine serum albumin (*m*/*v*). Next, membrane was incubated for 2 h at room temperature with horseradish-peroxidase-conjugated secondary antibodies specific to primary antibodies. Membranes were stained with ECL kit (Amersham Bioscience) according to manufacturer’s instructions and the protein bands were imaged with CAS-400SM Davinch-Chemi imager (Davinch-K).

### 4.7. Cellular Alkaline Phosphatase (ALP) Activity

ALP activity was evaluated in osteo-induced hBM-MSCs treated with or without samples. Cells were seeded in 6-well plates (1 × 10^6^ cells/well) and osteo-induced as previously described. At day 7 of differentiation, cells were lysed with 0.1% Triton X-100 and 25 mM carbonate buffer after washing with PBS. The cellular ALP activity was assessed using the supernatants of the cell lysates following 15 min centrifugation at 4 °C (12,000× *g*). The total protein content of the supernatant was determined by the Bradford protein determination method. Analysis of ALP activity was performed using a commercial kit (K412-500; BioVision, Hannover, Germany) according to the producer’s instructions.

### 4.8. Alizarin Red Staining

Calcified nodules formed by osteo-induced hBM-MSCs were observed by Alizarin Red staining. Cells were cultured in 6-well plates (1 × 10^6^ cells/well) and osteo-induced as previously described. At day 14 of differentiation, hBM-MSC osteoblasts were fixed on wells by addition of 70% EtOH (4 °C) after removing the medium. After 1 h, EtOH was removed from wells and the cells were washed with distilled water. Alizarin Red dying solution (pH 4.2, 2% *w*/*v*) was then added to each well (1 mL/well) and the plates were kept at room temperature for 10 min. After staining, wells were aspirated, and cells were washed with distilled water. Images of cells were taken by an Olympus microscope (Tokyo, Japan). Subsequently, the Alizarin Red dye was eluted from wells with 10% (*m*/*v*) cetylpyridinium chloride in 10 mM sodium phosphate buffer (Sigma-Aldrich, St. Louis, MO, USA) solution and mineralization was quantified by absorbance values at 560 nm using a Multiskan GO microplate reader (Tecan Austria GmbH).

### 4.9. Immunofluorescence Staining

Detection of perilipin-1, PPARγ and Wnt 10b expression in adipo-induced hBM-MSCs was observed by immunofluorescence staining. Cells were grown and induced differentiated on glass coverslips in same way previously noted. At day 10 of differentiation, cells were fixed and stained with anti-perilipin-1 (ab3526; Abcam) and anti-PPARγ antibody (ab9256; Abcam) conjugated with Alexa Fluor 488 (A-11008; Invitrogen), and ProLong Gold Antifade Reagent with DAPI (#8961; Cell Signaling Technology) for the nuclei highlighting. Fixation and staining of the cells were carried out using Immunofluorescence Application Solutions Kit (#12727; Cell Signaling Technology), according to the manufacturer’s instructions.

### 4.10. Statistical Analysis

Numerical results were given as an average of three independent experiments ± SD run in triplicate where applicable, unless otherwise noted. Groups in same data series were subjected to one-way analysis of variance (ANOVA) with post-hoc Duncan’s multiple range test for statistical analysis (SAS v9.1, SAS Institute, Cary, NC, USA) and differences were defined significant at *p* < 0.05 level.
